# Effectiveness of an interactive online group intervention based on pain neuroscience education and graded exposure to movement in breast cancer survivors with chronic pain: a randomised controlled trial

**DOI:** 10.1007/s00520-024-08887-4

**Published:** 2024-10-07

**Authors:** Patricia Martínez-Miranda, José Jesús Jiménez-Rejano, María Jesús Muñoz-Fernández, Cristina García-Muñoz, María Jesús Casuso-Holgado

**Affiliations:** 1 Departamento de Ciencias de la Salud y Biomédicas, Universidad Loyola de Andalucía, Seville, Spain; 2 CTS 1110, UMSS Research Group, Andalusia, Seville, Spain; 3 Department of Physiotherapy, Faculty of Nursing, Physiotherapy and Podiatry, Universidad de Sevilla, Seville, Spain; 4 Department of Physiotherapy, Avd. de los Cipreses S/N, University School Francisco Maldonado, 41640 Osuna, Spain; 5 Instituto de Biomedicina de Sevilla, IBiS, Departamento de Fisioterapia, Universidad de Sevilla, Seville, Spain

**Keywords:** Breast cancer, Quality of life, Pain neuroscience education, Graded exposure to movement, Therapeutic exercise, Yoga

## Abstract

**Purpose:**

To evaluate the effectiveness, compared with usual care, of an interactive online group programme combining pain neuroscience education (PNE) and graded exposure to movement (GEM) for improving quality of life and pain experience in breast cancer survivors with chronic pain.

**Methods:**

This single-blind randomised controlled trial included a sample of 49 breast cancer survivors who were randomly assigned to two groups (experimental: *n* = 22 and control: *n* = 27). The experimental group received a 12-week person-centred online programme based on pain neuroscience education and therapeutic yoga as gradual exposure to movement, while the control group continued with their usual care. The primary outcome was quality of life (FACT–B + 4); the secondary outcomes were related to the experience of chronic pain (pain intensity, pain interference, catastrophizing, pain self-efficacy, kinesiophobia, and fear avoidance behaviours). All variables were assessed at four time points (T0, baseline; T1, after PNE sessions; T2, after yoga sessions; T3, at 3-month follow-up). For data analysis, ANOVA (2 × 4) analysis of variance (95% CI) was used when outcomes were normally distributed. If not, within-group and between-group comparisons were calculated.

**Results:**

Thirty-six participants were included in the analysis (control group, 22; experimental group, 14). A significant time * group effect was observed in favour of the experimental group regarding the global quality of life score (*p* = 0.010, *η*_p_^2^ = 0.124). Significant differences in favour of the experimental group were observed for pain intensity, pain interference, catastrophizing, and pain self-efficacy. These differences persisted at follow-up.

**Conclusions:**

An online intervention based on PNE and GEM appears to be more effective than usual care for improving quality of life in breast cancer survivors with chronic pain, as a time per group interaction was reported. In addition, the intervention also significantly improved the participants’ experience of chronic pain. However, due to the study limitations further research is needed.

**Trial record:** NCT04965909 (26/06/2021).

## Introduction

Breast cancer is the most common type of cancer diagnosed in women [1], and it is estimated that the incidence of new cases will increase worldwide in the next decades. Survival rates are also increasing, but the survivorship phase is often associated with several cancer-related symptoms such as chronic pain, which can affect women’s quality of life and their social and professional reintegration [2–4]. As a result, there is an increasing demand for health care that addresses the chronic sequelae of cancer survivorship (e.g. chronic pain, fatigue). In addition, the side effects associated with prolonged use of pain medication make the development and improvement of non-pharmacological treatments essential [5].

Pain neuroscience education (PNE) is a cognitive-based intervention that aims to reconceptualise pain by explaining the neurophysiological mechanisms of pain and empowering people to manage their pain experience [6, 7]. PNE has reported broad benefits in addressing chronic pain in different populations, whether applied in isolation or as an adjuvant therapy [8–10], but it has been scarcely investigated in breast cancer [11–13]. González-Martín et al. [14] pointed out that PNE is an effective intervention for reducing pain intensity and the level of catastrophizing in patients with cancer pain, but no benefits were found in relation to quality of life. These authors, together with other previous reviews [14–16], highlighted the need for further studies investigating the benefits of patient pain education programmes based on a biopsychosocial content focused on the understanding of acute and chronic pain mechanisms, the identification of the key factors related to each individual painful experience or the relationship between pain, and our lifestyle habits, among others.

Therapeutic exercise is an important tool in the oncology field for improving quality of life, so it can be recommended as a therapy to be combined with patient education [17, 18]. In this line, graded exposure to movement (GEM) is a movement-based intervention that uses therapeutic exercise following the “twin peaks” metaphor proposed by Butler [6]. This metaphor attempts to symbolise how the gradual movement up to a painful baseline could help the system to progressively adapt and achieve more functionality with less pain. GEM has reported benefits in addressing chronic pain in several musculoskeletal conditions previously [19, 20], but it has not been investigated in the cancer population. In this clinical trial, yoga was applied following the basis of a graded exposure to movement intervention (GEM-Y), as yoga has been shown to be an effective exercise modality for improving quality of life in adults with cancer [21–23]. Furthermore, yoga is a mind–body exercise modality that allows us to follow a biopsychosocial approach [24]. To our knowledge, the combination of PNE with GEM-Y has never been studied in cancer previously. Therefore, the purpose of this clinical trial was to evaluate whether an interactive online group intervention combining PNE and GEM-Y is more effective than usual care in improving quality of life (primary outcome) and pain experience (secondary outcomes) in breast cancer survivors with chronic pain.

## Methodology

### Study design

A randomised controlled clinical trial was carried out according to the Consolidated Standards of Reporting Trials (CONSORT) Statement [25]. The Template for Intervention, Description and Replication Checklist (TIDieR) [26] was used as a guide to provide transparency and make the intervention replicable. The protocol of this study has been registered on clinicaltrials.org with the registry number NCT04965909.

### Protocol deviations

Only one deviation from the registered protocol needs to be reported. The method of data analysis was registered as an intention-to-treat analysis, but due to the adherence rates it was decided to perform a per-protocol analysis.

### Inclusion and exclusion criteria

The inclusion and exclusion criteria were developed following the PICOs model. Inclusion criteria are as follows: 1) women aged between 18 and 65 years; 2) diagnosis of stage 0–III breast cancer; 3) primary treatment (surgery, radiotherapy, and chemotherapy) completed at least 3 months ago but may still be receiving hormone therapy; 4) informed pain related to primary treatment in the last 6 months; 5) access to the Internet and an electronic device that allows the use of the applications used in this study and skills for their use or assistance from a close person who has them; 6) ability to communicate fluently verbally and in writing in the language of the research team (Spanish); and 7) approval to participate in the study by the coordinator of the health team that assisted during the course of cancer and its treatment. Exclusion criteria are as follows: 1) another previous type of cancer or breast cancer recurrence in a period of less than 1 year; 2) medical diagnosis of a neurological or autoimmune disease that limits or prevents exercise; 3) some type of pathology that is associated with a contraindication to physical exercise; and 4) the diagnosis of serious psychiatric or neurologic disorders that do not allow the participant to follow orders.

### Sampling method and sample’s size calculation

For sampling, non-probabilistic convenience and snowball methods were used. The sample size was calculated based on a previous study [27] with partial Eta2 effect size of 0.049 for the time * group interaction in the FACT-B score. Considering two groups, four measurements, a type I risk or *α* 0.05, type II risk or *β* 0.20 (study power of 80%), and an estimated dropout rate of 15%, a total of 40 participants (20 per group) are needed to be enrolled. Sample size was calculated using the G*Power software, version 3.1.9.7 (Heinrich-Heine University, Düsseldorf, Germany).

### Subjects’ recruitment

The sample for this study was recruited through the dissemination of the project using social networks (Facebook, Instagram) and with the collaboration of three Spanish breast cancer survivor support associations (Amama Sevilla, AGAMAMA, and ASAMMA). Participation in the study was voluntary and all participants were facilitated a written informed consent that must be signed to be part of the clinical trial.

### Group assignment and masking

For assignment, a random method was carried out using an online tool called ‘random allocation software’ (2.0 version). A stratified allocation was applied according to the women’s age (≤ 45 years old or > 45 years old). On each of the strata, a randomisation was carried out by blocks of constant size. The assignment sequence was hidden from the evaluator and the study subjects through an automated assignment system. The preparation of the sequence, the inclusion of the individuals in each group, and the assignment of the treatments were carried out by different members of the research team. On the other hand, the main researcher was blinded. Nonetheless, the physiotherapist and subjects were not blinded because of the type of intervention.

### Outcomes and data collection

The primary outcome of this trial was quality of life; secondary outcomes were related to chronic pain experiences: pain intensity and pain interference, catastrophizing level, pain self-efficacy, kinesiophobia, and fear-avoidance behaviours.

Quality of life was evaluated by the Spanish version of The Functional Assessment of Cancer Therapy—Breast plus arm morbidity (FACT–B + 4) [28]. It was originally validated by Brady et al. (1997) [29] and later the arm subscale was developed and incorporated into the existing FACT-B by Coster et al. (2001) [30]. It is a 41-item instrument designed to measure six domains of quality of life in breast cancer patients: physical well-being (PWB), social well-being (SWB), emotional well-being (EWB), functional well-being (FWB), breast-cancer subscale (BCS), and lymphedema (ARM) subscale. The overall score ranges from 0 to 148 points. A higher score translates into a better quality of life. The alpha coefficient (internal consistency) and the test–retest reliability of the Spanish version of the FACT-B + 4 were high (alpha = 0.87; intraclass correlation coefficient: 0.986) [29].

The Spanish version of the Modified Brief Pain Inventory—Short Form (BPI-SF) was used to assess pain intensity and pain interference with daily activities [31]. It is an 11-item instrument which has been previously assessed in the cancer population for this purpose [32]. The questionnaire has two subscales, one related to pain intensity (four items) and another related to the pain interference with activities of daily living (seven items). All items are scored on a scale from 0 to 10 and each dimension is calculated as an average, with a higher score indicating greater intensity or greater impact on daily life. The internal consistency and the test–retest reliability between dimensions were good (0.87 and 0.89) and low to moderate (0.53 and 0.77), respectively [31].

The Spanish version of the Pain Catastrophizing Scale was used to evaluate pain catastrophizing [33]. This scale is among the most valid instruments to assess this complex construct defined as “to view or present pain or pain-related problems as considerably worse than they actually are” [34]. The scale consists of three subscales (rumination, magnification, and helplessness), whose items will be valued from 0 (nothing) to 4 (all the time) to obtain a total score that ranges from 0 to 52. A higher score translates into a higher level of catastrophizing. The scale has adequate internal consistency (Cronbach’s alpha = 0.79), test–retest reliability (intraclass correlation coefficient = 0.84), and sensitivity to change (effect size ≥ 2) [33].

The Spanish version of the Pain Self-Efficacy Questionnaire (PSEQ) was chosen to assess self-efficacy level related to pain [35]. It is a 22-item instrument, and each item is scored from 0 to 10. 0 is equal to “I think I am totally incapable” and 10 is equal to “I think I am totally capable”. The total score ranges from 0 to 220. A higher score on the questionnaire corresponds to a higher level of self-efficacy. The internal consistency and the test–retest reliability between dimensions were 0.91 and 0.75) [35]. This measure has been previously used in cancer survivors with pain [36, 37].

The Tampa Scale for Kinesiophobia (TSK-11) Spanish version was chosen to assess the level of kinesiophobia [38]. This scale is one of the most used to evaluate kinesiophobia in patients with pain, including breast cancer population [32]. It is composed of two factors (avoidance of activity and harm) with a total of 11 items that are valued from 1 (totally disagree) to 4 (totally agree). The total score obtained ranges from 11 to 44. More punctuation shows a higher kinesiophobia level. The internal consistencies (Cronbach’s alpha = 0.79) found for this scale are good [38].

Finally, to fear-avoidance behaviours, we used the Fear Avoidance Components Scale Questionnaire—Spanish Version (FACS–SP) [39]. It is a questionnaire that allows us to evaluate a patient’s fear of pain and consequent avoidance of physical activity due to fear. The questionnaire consists of 20 items in which a patient rates his agreement with each statement on a 6-point Likert scale, where 0 = completely disagree, 6 = completely agree. There is a maximum score of 100. A higher score indicates more strongly held fear-avoidance beliefs. Five severity levels are available for clinical interpretation: subclinical (0–20), mild (21–40), moderate (41–60), severe (61–80), and extreme (81–100) [39]. It has been previously used in the breast cancer population [40].

In addition to these questionnaires, qualitative data were collected in an online interview. The information was collected through a semi-structured interview based on four pre-defined topics: pain experience (intensity, location, onset, evolution, factors that aggravate and relieve pain), pain coping strategies (e.g. analgesics, therapeutic exercise, physiotherapy), lifestyle habits (e.g. regular exercise or diet), and any notable milestones that might affect their pain experience (e.g. major work or family changes). The interviews were not recorded, but were transcribed. Responses were analysed inductively by one researcher (PMM), who identified similar themes for each topic. A weekly online diary was used to collect information on the acquisition of key concepts from the sessions. Finally, participants were asked about their satisfaction with the programme.

All outcomes and qualitative information were collected by two trained and blinded evaluators at four different timepoints: before intervention (T0), after 4-week PNE (T1), after 12-week complete intervention PNE + GEM-Y (T2), and after 3 months of follow-up (T3). Participants’ satisfaction was evaluated at T1 and T2. The outcomes were assessed using the above instruments that participants completed by themselves.

### Description of the intervention in the experimental and control group

**Fig. 1:**
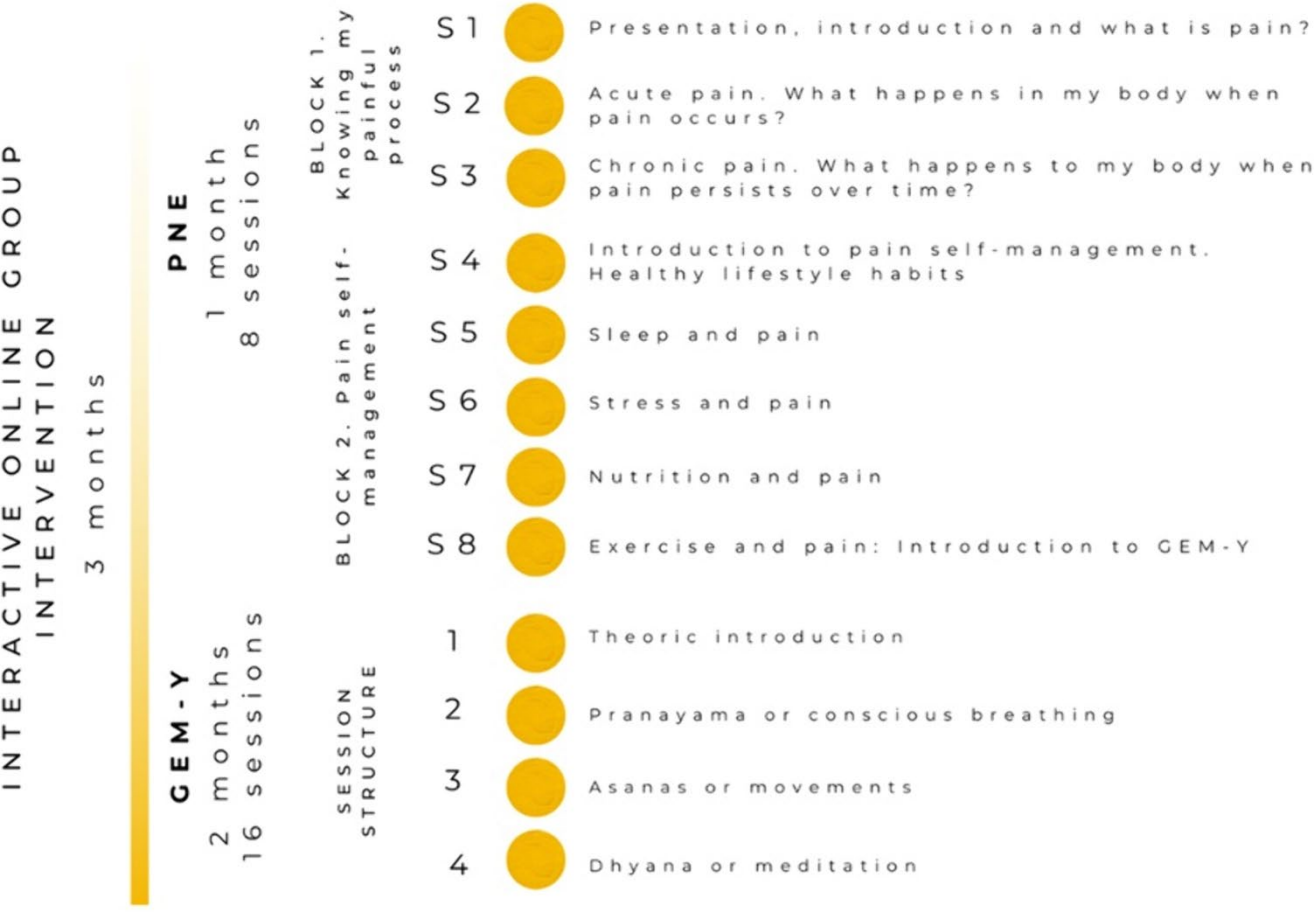
Structure of the intervention. GEM-Y, graded exposure to movement through therapeutic yoga; PNE, pain neuroscience education; S, session

An interactive online focused-person therapeutic programme, based on Rogers’ person-centred care approach [41] and combining PNE and GEM-Y, was implemented in the experimental group. All sessions were developed and supervised in-person by a trained physiotherapist using the videoconferencing platform of the University of Sevilla. In addition, WhatsApp and e-mail were used during the intervention to provide additional support, educational materials, or to answer queries. The sessions were applied in groups of 10–15 participants. The duration of the programme was 3 months, and it was divided into two parts. The first included 8 sessions of PNE during the first month (2 sessions per week, 1 h/session), and the second included 16 sessions of GEM-Y during the following 2 months (2 sessions per week, 1 h/session). Figure 1 summarises the structure of the intervention. PNE sessions focused on explaining the mechanisms of pain, explaining pain as an individual experience, and linking pain to lifestyle factors [6, 7]. A detailed description of the proposed intervention has been reported previously [42]. Attendance or non-attendance, with the reasons for non-attendance, was recorded for each session. In addition, a weekly individual online pain diary was used as a home-based work method. Participants in the control group did not receive any additional educational or movement-based intervention during the study period. They continued with their usual care for cancer-related symptoms and medical appointments. After the follow-up period, they were offered the content of the programme. An online educational booklet was provided to both groups. This booklet provided educational information in a very short format, addressing the following topics: breast cancer and its most common sequelae, biopsychosocial model, acute pain and chronic pain mechanisms, therapeutic exercise, and chronic pain.

### Method for data analysis

The software IBM Statistics Package for Social Science®, v.29 (IBM Corp, NY, USA) was used to perform the statistical processing of data following a per protocol analysis. It was established that only the data of those participants in the experimental group who had attended at least 50% of the sessions would be analysed (per protocol analysis). The normal distribution of the variables was assessed with the Shapiro–Wilk test. Descriptive data are reported as mean ± standard deviation, or median (interquartile range Q3–Q1). For the variables where the four measurements followed a normal distribution, a mixed factorial ANOVA was used with group as the between-subject factor and time as the within-subject factor (partial Eta squared coefficient ηp2 effect size). Prior to ANOVA analysis, the Mauchly test was used to check the sphericity assumption; if the sphericity hypothesis was not met, the Greenhouse–Geisser correction was used. In addition, for variables where no normal distribution was observed at any time point, comparisons within and between groups were assessed using the Student *t* test/Welch *t* test (Cohen’s *d* effect size) or the Mann–Whitney *U* test (Rosenthal’s *r* effect size) with Bonferroni corrections. All statistical tests were performed considering a confidence interval (hereinafter CI) of 95% (*p*-value < 0.05).

## Results

**Fig. 2:**
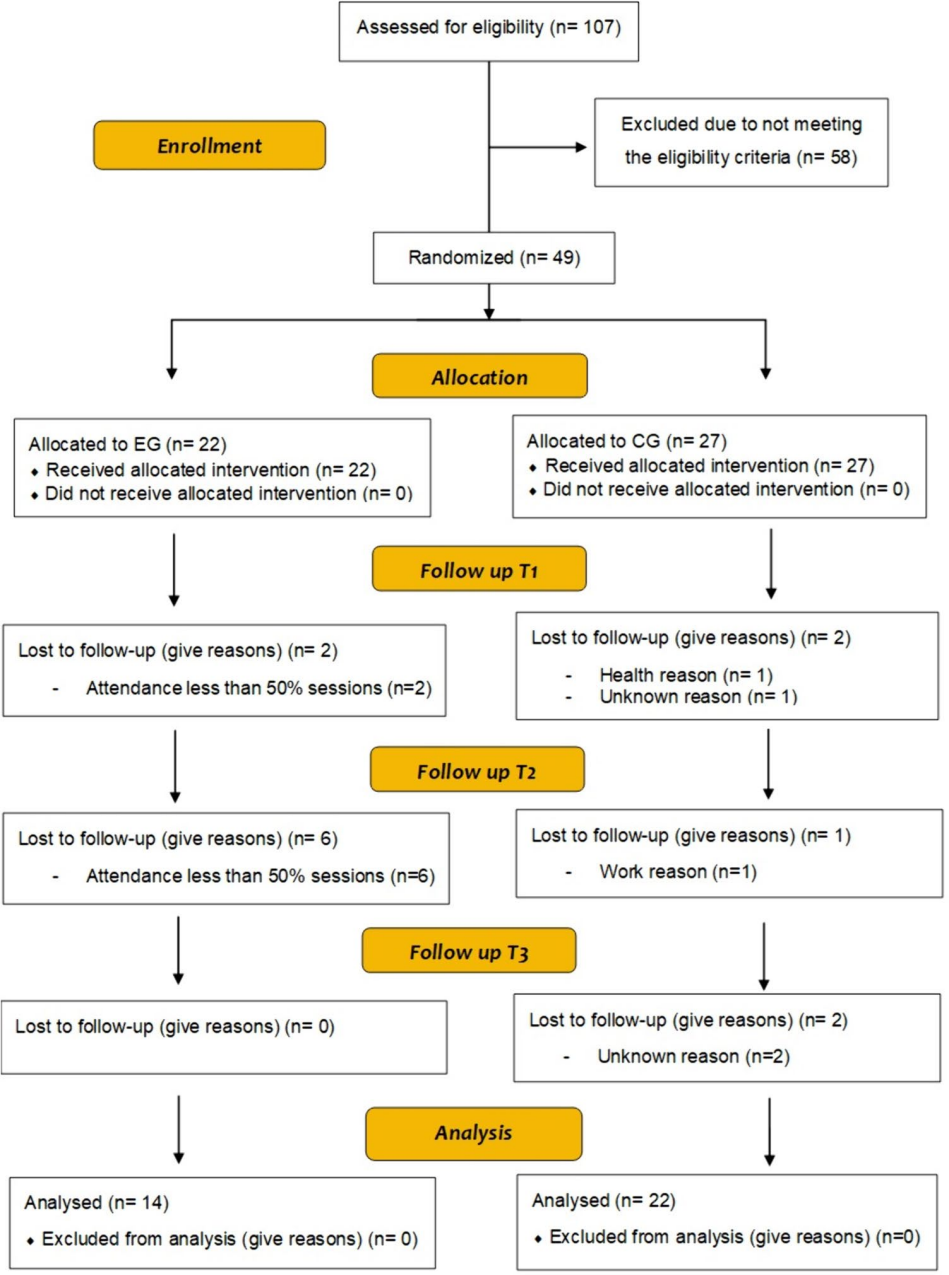
CONSORT flow diagram

A total of 107 breast cancer survivors were recruited, of which 58 did not meet the selection criteria, obtaining a sample of 49 women (27 were randomly assigned to the control group and 22 to the experimental group). Five participants drop out in the control group and eight in the experimental group. The reasons for dropout were all related to the difficulty of fitting the intervention schedule around other responsibilities in daily life (e.g. work, family, medical). Finally, a total of 36 participants were analysed (22 in the control group and 14 in the experimental group). No adverse or harmful events were reported in either group. The flow diagram of the trial is presented in Fig. 2. Baseline characteristics for each group are shown in Table 1.

**Table 1:** Baseline characteristics of the sample

**Variable (tool)**	**CG**	**EG**
(score range)	(*n* = 22)	(*n* = 14)
	M (SD)	
**Age (years)**	50.00 (8.04)	49.21 (5.91)
**Quality of life (FACT-B + 4)** **Overall score** (0–148)	66.01 (15.01)	75.43 (16.12)
**Quality of life (FACT-B + 4)** **PWB** (0–28)	16.64 (5.07)	18.50 (6.12)
**Quality of life (FACT-B + 4)** **SWB** (0–28)	15.96 (6.97)	18.93 (4.97)
**Quality of life (FACT-B + 4)** **EWB** (0–24)	14.05 (5.28)	15.71 (3.87)
**Quality of life (FACT-B + 4)** **FWB** (0–28)	15.55 (4.16)	17.21 (4.78)
**Quality of life (FACT-B + 4)** **BCS** (0–40)	18.23 (7.24)	20.79 (5.32)
**Quality of life (FACT-B + 4)** **ARM** (0–20)*	10.68 (4.47)	12.29 (3.41)
**Pain intensity (BPI-SF)** (0–10)	4.16 (1.69)	3.70 (2.03)
**Pain interference (BPI-SF)** (0–10)	4.21 (1.84)	3.64 (2.77)
**Kinesiophobia level (TSK-11)** (11–44)	25.68 (6.22)	22.79 (6.32)
**Catastrophizing level (PCS)** (0–52)	18.91 (12.68)	14.36 (9.94)
**Fear—avoidance behaviours (FACS-SP)** (0–100)	43.36 (23.01)	34.79 (18.36)
**Self-efficacy level (PSEQ)** (0–220)	126.14 (37.43)	131.86 (31.72)

*This subscale is not included in the calculation of the overall score of the FACT-B + 4

*BPI-SF* Brief Pain Inventory—Short Form, *CG* control group, *EG* experimental group, *FACS-SP* Fear of Pain Avoidance Components Questionnaire—Spanish Version, *FACT-B* + *4* Functional Evaluation for Cancer Treatment—Breast Cancer, *M* mean, *PCS* Pain Catastrophizing Scale, *PSEQ* Pain Self-Efficacy Questionnaire, *SD* standard deviation, *TSK-11* Tampa Scale for Kinesiophobia.

### *Primary outcome: quality of life (FACT—B* + *4)*

Tables 2 and 3 show the results of the primary outcome. The mixed factorial ANOVA analysis revealed a significant time*group interaction for the overall quality of life score (*F* (3, 102) = 4.80, *p* = 0.010; *η*_p_^2^ = 0.124), but not for the physical, functional, and breast cancer subscales (Table 2). A significant difference in favour of the experimental group was also observed for the emotional subscale at the follow-up assessment, but not for any of the other FACT-B + 4 dimensions (Table 3).

**Table 2:** Results of mixed factorial ANOVA for variables following a normal distribution

Variable		Group			T1 M (SD)		T2 M (SD)			T3 M (SD)		
Quality of life (FACT-B + 4) Overall score		CG (*n* = 22)			65.50 (13.73)		79.95 (20.68)			80.73 (21.22)		
		EG (*n* = 14)			83.62 (14.71)		101.71 (20.83)			104.50 (20.59)		
		Between group difference, MD (CI al 95%)			** − 18.12** **(− 27.92; − 8.31)** ***p* = 0.001 *d* = 1.28**		** − 21.76** **(− 36.17; − 7.35)** ***p* = 0.04 *d* = 1.05**			** − 23.77** **(− 38.35; − 9.19) *p* = 0.002 *d* = 1.13**		
		MD (CI 95%)		CG (*n* = 22)	T0 vs T1	T0 vs T2		T0 vs T3	T1 vs T2		T1 vs T3	T2 vs T3
					0.51 (− 5.76; 6.78) *p* = 0.999	** − 13.95** **(− 22.34;** ** − 5.55)** ***p* < 0.001**		** − 14.73** **(− 23.65; − 5.79)** ***p* < 0.001**	** − 14.46** **(− 21.19;** ** − 7.72)** ***p* < 0.001**		** − 15.23** **(− 22.28; − 8.18)** ***p* < 0.001**	− 0.77 (− 5.53; 3.99) *p* = 0.999
				EG (*n* = 14)	T0 vs T1	T0 vs T2		T0 vs T3	T1 vs T2		T1 vs T3	T2 vs T3
					** − 8.19** **(− 16.05;** ** − 0.33)** ***p* = 0.037**	** − 26.29** **(− 36.81; − 15.76)** ***p* < 0.001**		** − 29.07** **(− 40.27; − 17.87)** ***p* < 0.001**	** − 18.10** **(− 26.53; − 9.66)** ***p* < 0.001**		** − 20.88** **(− 29.72; − 12.04)** ***p* < 0.001**	− 2.79 (− 8.75; 3.18) *p* = 0.999
	ANOVA	Time * group interaction			***F***_**(3, 102)**_** = 4.80; *p* = 0.010*η***_**p**_^**2**^** = 0.12**							
		Intersubject factor group			***F***_**(1, 34)**_** = 10.45; *p* = 0.003*η***_**p**_^**2**^** = 0.235**							
		Within-subject factor			***F***_**(3, 102)**_** = 59.30; *p* < 0.001*η***_**p**_^**2**^** = 0.636**							
Quality of life (FACT-B + 4) Physical subscale (PWB)		CG (*n* = 22)			16.45 (4.76)		15.50 (4.44)			15.91 (5.19)		
		EG (*n* = 14)			21.64 (3.73)		20.07 (4.39)			21.07 (3.89)		
		Between group difference, MD (CI al 95%)			** − 5.19 (− 8.24; − 2.14)** ***p* = 0.002 *d* = 1.18**		** − 4.57 (− 7.64; − 1.50)** ***p* = 0.005 *d* = 1.03**			** − 5.16 (− 8.45; − 1.87)** ***p* = 0.003 *d* = 1.09**		
		MD (CI 95%)	CG (*n* = 22)		T0 vs T1	T0 vs T2		T0 vs T3	T1 vs T2		T1 vs T3	T2 vs T3
					0.18 (− 1.85; 2.22) *p* = 0.999	1.14 (− 1.05; 3.32) *p* = 0.923		0.73 (− 2.33; 3.78) *p* = 0.999	0.96 (− 0.83; 2.74) *p* = 0.855		0.55 (− 1.97; 3.06) *p* = 0.999	− 0.41 (− 2.30; 1.48) *p* = 0.999
			EG (*n* = 14)		T0 vs T1	T0 vs T2		T0 vs T3	T1 vs T2		T1 vs T3	T2 vs T3
					− 3.14 (− 5.70; − 0.59) *p* = 0.054	− 1.57 (− 4.31; 1.17) *p* = 0.701		− 2.57 (− 6.40; 1.26) *p* = 0.412	1.57 (− 0.66; 3.80) *p* = 0.341		0.57 (− 2.58; 3.72) *p* = 0.999	− 1.00 (− 3.37; 1.37) *p* = 0.999
	ANOVA	Time * group interaction			*F*_(3, 102)_ = 2.93; *p* = 0.056 *η*_p_^2^ = 0.079							
		Intersubject factor group			***F***_**(1, 34)**_** = 8.67; *p* = 0.006*η***_**p**_^**2**^** = 0.203**							
		Within-subject factor			*F*_(3, 102)_ = 2.14; *p* = 0.121 *η*_p_^2^ = 0.059							
Quality of life (FACT-B + 4) Functional subscale (FWB)		CG (*n* = 22)			15.14 (4.50)		15.95 (4.63)			15.45 (4.72)		
		EG (*n* = 14)			18.71 (4.53)		19.43 (4.91)			20.36 (4.27)		
		Between group difference, MD (CI al 95%)			** − 3.58 (− 6.71; − 0.45)** ***p* = 0.026 *d* = 0.79**		** − 3.47 (− 6.77; − 0.18)** ***p* = 0.039 *d* = 0.73**			** − 4.90 (− 8.07; − 1.74)** ***p* = 0.003 *d* = 1.08**		
		MD (CI 95%)	CG (*n* = 22)		T0 vs T1	T0 vs T2		T0 vs T3	T1 vs T2		T1 vs T3	T2 vs T3
					0.41 (− 1.41; 2.23) *p* = 0.999	− 0.41 (− 2.57; 1.75) *p* = 0.999		0.09 (− 1.88; 2.06) *p* = 0.999	− 0.82 (− 2.83; 1.20) *p* = 0.999		− 0.32 (− 2.47; 1.83) *p* = 0.999	0.50 (− 1.10; 2.10) *p* = 0.999
			EG (*n* = 14)		T0 vs T1	T0 vs T2		T0 vs T3	T1 vs T2		T1 vs T3	T2 vs T3
					− 1.50 (− 3.78; 0.78) *p* = 0.443	− 2.21 (− 4.92; 0.49) *p* = 0.169		** − 3.14** **(− 5.61;** ** − 0.67)** ***p* = 0.007**	− 0.71 (− 3.24; 1.81) *p* = 0.999		− 1.64 (− 4.34; 1.05) *p* = 0.580	− 0.93 (− 2.94; 1.08) *p* = 0.999
	ANOVA	Time * group interaction			*F*_(3, 102)_ = 2.80; *p* = 0.051 *η*_p_^2^ = 0.076							
		Intersubject factor group			***F***_**(1, 34)**_** = 5.94;*p* = 0.020*η***_**p**_^**2**^** = 0.149**							
		Within-subject factor			***F***_**(3, 102)**_** = − 3.14;*p* = 0.035*η***_**p**_^**2**^** = 0.085**							
Quality of life (FACT-B + 4)—Breast cancer subscale (BCS)		CG (*n* = 22)			18.09 (4.19)		17.45 (6.05)			18.09 (5.14)		
		EG (*n* = 14)			23.07 (4.83)		24.00 (7.77)			25.14 (6.61)		
		Between group difference, MD (CI al 95%)			** − 4.98 (1.52)** ***p* = 0.002 *d* = 1.12**		** − 6.55 (2.31)** ***p* = 0.008 *d* = 0.97**			** − 7.05 (1.96)** ***p* = 0.001 *d* = 1.05**		
		MD (CI 95%)	CG (*n* = 22)		T0 vs T1	T0 vs T2		T0 vs T3	T1 vs T2		T1 vs T3	T2 vs T3
					0.14 (− 2.89; 3.16) *p* = 0.999	0.77 (− 3.03; 4.58) *p* = 0.999		0.14 (− 3.48; 3.75) *p* = 0.999	0.64 (− 2.04; 3.31) *p* = 0.999		0.00 (− 2.05; 2.05) *p* = 0.999	− 0.64 (− 2.68; 1.41) *p* = 0.999
			EG (*n* = 14)		T0 vs T1	T0 vs T2		T0 vs T3	T1 vs T2		T1 vs T3	T2 vs T3
					− 2.29 (− 6.08; 1.51) *p* = 0.602	− 3.21 (− 7.98; 1.55) *p* = 0.405		− 4.36 (− 8.89; 0.18) *p* = 0.065	− 0.93 (− 4.28; 2.42) *p* = 0.999		− 2.07 (− 4.64; 0.49) *p* = 0.181	− 1.14 (− 3.71; 1.42) *p* = 0.999
	ANOVA	Time * group interaction			*F*_(3, 102)_ = 2.86, *p* = 0.063; *η*_p_^2^ = 0.078							
		Intersubject factor group			***F***_**(1, 34)**_** = 9.10, *p* = 0.005;*η***_**p**_^**2**^** = 0.211**							
		Within-subject factor			*F*_(3, 102)_ = 2.10,*p* = 0.129; *η*_p_^2^ = 0.058							

Significant differences in bold (*p* < 0.05).

*BCS* breast-cancer subscale, *CI* confidence interval, *CG* control group, *d* Cohen´s *d*, *EG* experimental group, *FACT-B* + *4* Functional Evaluation for Cancer Treatment—Breast Cancer, *FWB* functional well-being, *M* mean, *MD* mean difference, *η*_*p*_^*2*^ partial eta square, *PWB* physical well-being, *SD* standard deviation, *T0* before intervention, *T1* assessment after PNE block, *T2* assessment after PNE + GEM-Y, *T3* assessment after 3-month follow-up.

**Table 3:** Results of the primary outcome subscales that did not follow a normal distribution: within-group and between-group comparisons

**Variable**		**CG** ***n* = 22** *M* (SD)/median (IQR)	**EG** ***n* = 14** *M* (SD)/median (IQR)	**MD (SE)** ***p*-value effect size**
**Quality of life** **(FACT-B + 4)** **Social subscale** **(SWB)**	**T1**	15.82 (6.42)	20.19 (5.77)	− 4.37 (2.11) *p* = 0.138^c^ *d* = 0.71
	**T2**	16.55 (6.71)	20.00 (5.74)	− 4.37 (2.11) *p* = 0.363^c^ *d* = 0.54
	**T3**	18.00 (13.00–21.00)^a^	19.50 (12.00–25.00)^a^	*p* = 0.999^c^ *r* = 0.14
**Quality of life (FACT-B + 4)** **Emotional subscale** **(EWB)**	**T1**	13.59 (5.06)	16.86 (3.70)	− 3.27 (1.57) *p* = 0.135^b^ *d* = 0.71
	**T2**	14.50 (11.00–19.00)^a^	**19.50 (15.00–21.00)**^**a**^	*p* = 0.141^c^* r* = 0.33
	**T3**	14.36 (5.45)	**18.57 (3.94)**	**− 4.21 (1.68)** ***p* = 0.033**^**b**^***d* = 0.85**
**Quality of life (FACT-B + 4)** **Lymphedema subscale** **(ARM)**	**T1**	10.73 (4.03)	13.50 (3.67)	− 2.77 (1.33) *p* = 0.135^b^ *d* = 0.71
	**T2**	11.50 (7.00–14.00)^a^	**14.00 (11.00–17.00)**^**a**^	*p* = 0.339^c^ *r* = 0.26
	**T3**	**12.50 (4.42)**	13.43 (3.72)	− 0.93 (1.42) *p* = 0.999^b^ *d* = 0.22

^a^Median (IQR)

^b^t-Student test

^c^Mann-Whitney U test; within-group and between-group significant differences in bold (*p* < 0.05)

*CG* control group, *EG* experimental group, *FACT-B* + *4* Functional Evaluation for Cancer Treatment—Breast Cancer, *IQR* interquartile range, *M* mean, *MD* mean difference, *SE* standard error, *SD* standard deviation, *T1* assessment after PNE block, *T2* assessment after PNE + GEM-Y, *T3* assessment after 3-month follow-up.

### Secondary outcomes: pain intensity (BPI), pain interference (BPI), kinesiophobia (TSK-11), catastrophizing level (PCS), fear—avoidance behaviours (FACS-SP), and self-efficacy (PSEQ)

Table 4 shows the results of the secondary outcomes. Significant differences in favour of the experimental group were found for pain intensity (*p* = 0.004, *d* = 1.44), pain interference (*p* < 0.001, *d* = 2.08), catastrophizing level (*p* = 0.039, *r* = 0.41), and pain self-efficacy (*p* = 0.009, *r* = 0.50). These differences persisted at follow-up (T3). No differences were found for kinesiophobia or fear-avoidance behaviours.

**Table 4:** Results of the secondary outcomes that did not follow a normal distribution: within-group and between-group comparisons

**Variables**		**CG** ***n* = 22** *M* (SD)/median (IQR)	**EG** ***n* = 14** *M* (SD)/median (IQR)	**MD (SE)** ***p*-value effect size**
**Pain intensity** **(BPI-SF)**	**T1**	4.85 (1.53)	2.39 (1.72)	**2.46 (0.55)** ***p* < 0.001**^**a**^***d* = 1.53**
	**T2**	5.40 (1.46)*	2.59 (2.54)	**2.81 (0.75)** ***p* = 0.004**^**b**^***d* = 1.44**
	**T3**	4.88 (40.00–6.25)^c^	1.13 (0.00–2.25)^c^	***p* < 0.001**^**d**^***r* = 0.61**
**Pain interference** **(BPI-SF)**	**T1**	5.50 (4.43–6.57)^c^*	1.29 (0.57–4.71)^c^	***p* = 0.001**^**d**^***r* = 0.53**
	**T2**	5.25 (1.77)*	1.65 (1.66)*	**3.60 (0.59)** ***p* < 0.001**^**a**^***d* = 2.08**
	**T3**	5.29 (2.86–6.00)^c^	0.57 (0.29–1.86)^c^*	***p* < 0.001**^**d**^***r* = 0.58**
**Kinesiophobia level** **(TSK-11)**	**T1**	35.14 (15.94)	20.57 (20.35)	14.57 (6.07) *p* = 0.066^a^ *d* = 0.82
	**T2**	24.50 (21.00–28.00)^c^	20.50 (18.00–23.00)^c^	*p* = 0.096^d^ *r* = 0.36
	**T3**	31.55 (17.22)	16.00 (19.41)*	15.55 (6.18) *p* = 0.066^a^ *d* = 0.86
**Catastrophizing level** **(PCS)**	**T1**	18.86 (9.64)	10.64 (8.27)	**8.22 (3.13)** ***p* = 0.039**^**a**^***d* = 0.90**
	**T2**	15.50 (8.00–26.00)^c^	4.00 (1.00–11.00)^c^	***p* = 0.039**^**d**^***r* = 0.41**
	**T3**	15.00 (5.00–28.00)^c^	2.50 (0.00–11.00)^c^*	***p* = 0.009**^**d**^***r* = 0.50**
**Fear-avoidance behaviours** **(FACS-SP)**	**T1**	42.55 (19.86)	25.64 (13.68)	**16.90 (6.07)** ***p* = 0.027**^**a**^***d* = 1.03**
	**T2**	37.50 (22.00–51.00)^c^	18.50 (10.00–38.00)^c^*	*p* = 0.114^d^ *r* = 0.35
	**T3**	35.77 (22.80)	21.07 (20.31)*	14.70 (7.48) *p* = 0.174^a^ *d* = 0.67
**Self-efficacy level** **(PSEQ)**	**T1**	131.05 (24.66)	141.50 (26.72)	− 10.46 (8.71) *p* = 0.714^a^ *d* = 0.41
	**T2**	123.00 (95.00–140.00)^c^	159.00 (151.00–169.00)^c^*	***p* = 0.009**^**d**^***r* = 0.50**
	**T3**	126.00 (84.00–150.00)^c^	157.00 (149.00–182.00)^c^*	***p* = 0.045**^**d**^***r* = 0.41**

^a^t-Student test

^b^t-Welch test

^c^Median, 1st–3rd quartiles

^d^Mann-Whitney U test

*Within-group significant difference (*p* ≤ 0.05); between-group significant differences in bold (*p* ≤ 0.05)

*BPI-SF* Brief Pain Inventory—Short Form, *CG* control group, *EG* experimental group, *FACS-SP* Fear of Pain Avoidance Components Questionnaire—Spanish Version, *IQR* interquartile range, *M* mean, *PCS* Pain Catastrophizing Scale, *PSEQ* Pain Self-Efficacy Questionnaire, *SD* standard deviation, *TSK-11* Tampa Scale of Kinesiophobia, *T1* assessment after PNE block, *T2* assessment after PNE + GEM-Y, *T3* assessment after 3-month follow-up.

### Secondary outcomes: qualitative data analysis

The main themes that emerged from the interviews were prolonged rest and overexertion (overdoing exercise) as factors that aggravate pain; movement and well-dosed exercise as factors that relieve pain; and regular exercise as a lifestyle habit that improves the experience of pain. The analysis of the pain diaries showed that the acquisition of the key concepts of the weekly PNE sessions was higher than 75%, except for the content of week 3, where it was slightly lower. Regarding the yoga sessions, almost 100% of participants perceived an improvement in their pain-related functionality during the GEM-Y.

Overall, participants’ satisfaction with the programme was quite good, with most participants saying they were “very satisfied”. The most highly rated aspects were the usefulness of the programme, the practical content, and the quality of the explanations. The aspects that received a lower average score were the group and online work experience.

## Discussion

This clinical trial aimed to evaluate the effectiveness of an interactive online group intervention combining PNE and GEM-Y in improving the quality of life (primary outcome) and pain experience (secondary outcomes) in breast cancer survivors with chronic pain, compared with usual care. The results showed a significant time per group interaction in favour of the experimental group for the overall quality of life measure. In addition, the intervention appears to be more effective than control for pain experience outcomes, except for the level of kinesiophobia, as significant differences were observed immediately after intervention (T2) and at the follow-up period (T3).

Our results are in contrast with previous research on the effectiveness of pain education in improving quality of life in people with cancer-related pain. González-Martín et al. [14] conducted a systematic review on this topic and concluded that patient education did not improve quality of life in patients with cancer. However, our research has demonstrated a significant time per group interaction in favour of the experimental group after a 4-week PNE intervention for the overall quality-of-life score of the FACT-B + 4. In addition, our results for the total score are clinically relevant as the between-group difference in their within-group score changes (T0–T2) is above the minimum clinically important difference for this measure [43]. This controversy with previous research could be explained by the fact that some of the pain education interventions studied were based on a more general concept of pain education, which was not in line with the principles of PNE [6, 7]. In our case, a patient-centred approach was followed and our content was based on a biopsychosocial approach.

Regarding pain experience outcomes, our results are partially consistent with previous research. We observed significant differences in favour of the experimental group across all assessment timepoints for pain intensity, pain interference, and catastrophizing. Similarly, Pas et al. (2020) [11] conducted a pilot study on the effect of PNE on persistent pain in breast cancer survivors and concluded that PNE seemed to have a beneficial effect in improving pain intensity and level of catastrophizing in this population. However, when PNE is used as a preventive strategy for chronic pain, the results are controversial. Manfuku et al. (2021) [12] observed that perioperative PNE was more beneficial than general biomedical information for the prevention of pain chronification after breast cancer surgery, while Dams et al. (2022) [13] concluded that preoperative PNE had no significant effects on pain-related disability or pain intensity 18 months after surgery. Finally, González-Martín et al. [14] concluded that PNE has a positive effect on pain intensity and kinesiophobia, which is partially supported by our results, since we observed important benefits on pain intensity and pain interference, but the level of kinesiophobia reported by the participants did not change after the PNE block or the GEM-Y sessions. In general, although PNE interventions seem to have a positive effect on reducing pain intensity in cancer-related pain, the limitations of previous research on this topic and our own force us to interpret these findings with caution.

Our findings regarding the effect of yoga on quality of life are consistent with previous studies that support the recommendation of yoga as a therapeutic intervention to improve quality of life in breast cancer [23, 44–46]. In contrast, the effects of yoga on cancer-related pain are controversial [44, 46]. However, we found important benefits for chronic pain, as pain intensity improved after the yoga intervention, and pain interference and pain self-efficacy only improved when PNE was used in conjunction with the GEM-Y intervention. This discrepancy with previous research could be explained by the fact that our intervention, unlike traditional yoga interventions, placed particular emphasis on delivering it according to the principles of a GEM intervention, following the “twin peaks” metaphor explained previously [6], so that the progression of the yoga exercises was directly related to the evolution of the individual’s pain experience.

The relevance of results of this study should be interpreted considering some methodological limitations and strengths. Some participants (*n* = 8) failed to attend sessions, which forced us to develop a per-protocol analysis and resulted in a smaller sample size in the experimental group. In addition, the reasons for non-attendance were related to the difficulty of fitting two sessions per week over 12 weeks with other daily responsibilities, so a shorter intervention may have been more appropriate in this population. Thirdly, a follow-up period of 3 months could be considered short; however, when educational interventions are presented in an online modality, it is common to consider this time period [47]. Finally, the proposed snowball sampling method could limit the generalisability of our results, as well as the representativeness of the subjects analysed.

To the best of our knowledge, this is the first clinical trial to combine PNE and GEM-Y in breast cancer population. Secondly, the online modality had some advantages such as the accessibility of the program regardless of the participant’s location or the reduction of costs and resources [48]. In addition, the choice of this modality was based on the results of a previous review [47], which indicated that the mixed format of patient education (i.e. face-to-face meetings plus online information) was the most beneficial for improving quality of life in breast cancer survivors [47]. These findings lead us to hypothesise that an online educational intervention with direct person-therapist interaction could be a more contemporary version of the mixed format, supported by new technologies.

## Conclusions

A 12-week interactive online group intervention based on PNE plus GEM-Y appears to be more effective than usual care in improving quality of life in breast cancer survivors with chronic pain. A time per group interaction was observed for the FACT-B + 4 overall quality-of-life score. The intervention also appears to be more effective than usual care for improving the participants’ pain experiences. Most of the effects of the intervention were maintained at the 3-month follow-up. However, due to the study limitations, these res ults must be interpreted with caution and further research is needed.

## Data Availability

The data that support the findings of this study are not openly available due to reasons of sensitivity and are available from the corresponding author upon reasonable request. Data are located in controlled access data storage at Karolinska Institutet/University of Seville.
